# Preparation and *In Vitro* Evaluation of Glycyrrhetinic Acid-Modified Curcumin-Loaded Nanostructured Lipid Carriers

**DOI:** 10.3390/molecules19022445

**Published:** 2014-02-21

**Authors:** Yang Chu, Dan Li, Yi-Fan Luo, Xiao-Jin He, Ming-Yan Jiang

**Affiliations:** 1 Department of Pharmacy, The First Affiliated Hospital of China Medical University, 155 Nanjing Street, Shenyang 110001, China; 2 Department of The First Clinical Pharmacy, China Medical University, 92 Bei’er Road, Shenyang 110001, China; 3 Department of Pharmacy, Central Hospital Attached to Shenyang Medical College, No. 5 the 7th south-west Road, TieXi district, Shenyang 110024, China; 4 School of Pharmacy, Shenyang Pharmaceutical University, 103 Wenhua Street, Shenyang 110016, China

**Keywords:** curcumin, glycyrrhetinic acid, nanostructured lipid carriers, anti-HepG_2_

## Abstract

Curcumin, a phenolic antioxidant compound derived from the rhizome of the turmeric plant *Curcuma longa*, has proven to be a modulator of intracellular signaling pathways that control cancer cell growth, inflammation, invasion and apoptosis, revealing its anticancer potential. In this study, a Glycyrrhetinic Acid-Modified Curcumin-Loaded Nanostructured Lipid Carrier (Cur-GA-PEG-NLC) was prepared by the film ultrasound method to improve the tumor-targeting ability. The drug content was detected by an UV spectrophotometry method. The encapsulation efficiency of curcumin in the nanostructured lipid carriers (NLCs) was determined using a mini-column centrifugation method. The encapsulation efficiency for various Cur-GA-PEG-NLC was within the range of 90.06%–95.31% and particle size was between 123.1 nm and 132.7 nm. An *in vitro* MTT assay showed that Cur-GA10%-PEG-NLC had significantly high cellular uptake and cytotoxicity against HepG2 cells compared with other groups.

## 1. Introduction

Cancer is leading cause of death in the World. Current chemotherapeutic agents such as alkylating agents, mustards, anti-metabolites, spindle poisons, and DNA binders and cutters, target a specific biological pathway, which ultimately shrinks tumor size, but often fails to eradicate tumors or prevent their recurrence. It was reported that repeated treatment with these agents results in tumors resistant to chemotherapy, so it is crucial to identify natural products that might have growth inhibitory and apoptosis induction properties in human cancer cells and without toxicity issues towards normal cells [1]. On the basis of its high pharmacological effect and low toxicity, curcumin might be a potential drug candidate for the treatment of cancer.

**Figure 1:**
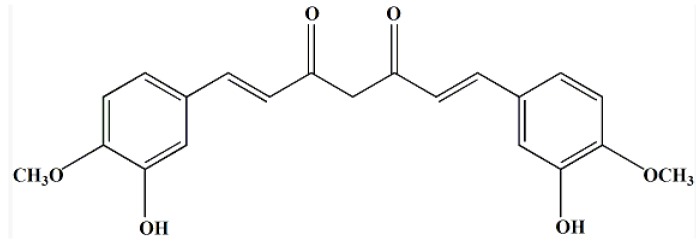
Structure of curcumin.

Curcumin (Figure 1) is a phenolic antioxidant compound derived from the rhizome of the turmeric plant *Curcuma longa*. Curcumin is widely used as colorant and dye in the food field [2]. Curcumin is also a prominent anti-inflammation, anti-toxin, anti-cystic fibrosis, anti-Alzheimer’s, hepatoprotective and anti-malarial agent, in addition to its anti-cancer properties [3,4,5,6,7,8,9]. Many studies have shown that curcumin is a potent inhibitor of arachidonic acid metabolism and cyclooxygenase activity in epidermis and intestinal mucosa [10,11,12,13,14]. Recent studies had found that curcumin inhibited proliferation of Sk-hep-1 cells with a dose-effect relationship. Cell cycle analysis results showed that curcumin treatment resulted in dramatic accumulation of Sk-hep-1 cells at the G0/G1 or G2/M phase, which indicated that curcumin was able to inhibit proliferation and induce apoptosis in Sk-hep-1 cells caused by downregulating the expression of MDR1 mRNA [15]. Because of its good anticancer effects the USA National Cancer Institute (NCI) had listed it as a third generation cancer chemo-preventive drug [16].

Nanostructured lipid carriers (NLCs) are a new type of drug delivery system generated from solid lipid nanoparticles (SLNs), which offer some benefits in contrast to other colloidal carrier schemes. The solid lipid carrier is a natural or synthetic lipid such as lecithin or triacylglycerol. The liquid oil is dropped into the solid lipid to make nanoparticles with crystalline or amorphous structures. The drug is wrapped in the lipid nucleus and the particle size is between 50–1,000 nm. NLCs show a higher loading capability for compounds and also have a lower water content in their elemental suspensions and a less inclination to unpredictable gelation [17,18,19]. NLCs provide a controlled pharmaceutical form and an increase in chemical stability of the incorporated drugs. Furthermore, NLCs are a protected carrier which can be produced effortlessly on a large scale [17,20,21,22,23].

**Figure 2:**
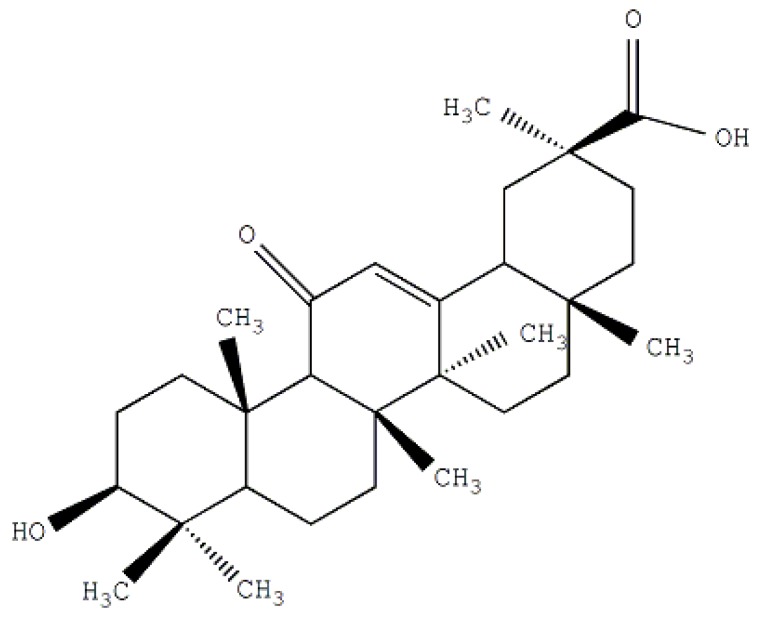
Structure of glycyrrhetinic acid (GA).

Glycyrrhetinic acid (GA) is a hydrolysis product of glycyrrhizic acid (GL) which is the main components of the Traditional Chinese Medicine liquorice. The structure of glycyrrhetinic acid is shown in Figure 2. It was reported that there are some highly specific GA binding sites located on the surface of liver parenchyma cells. Some researchers also reported that liposomes modified with GA expressed a considerably high affinity to hepatocytes [24,25,26]. GA conjunction with NLC could produce a smart system with lower toxicity and higher bioactivity. The investigations of GA-mediated targeted drug delivery systems such as liposomes, nanoparticles and polymeric micelles have become more and more popular. In our study, curcumin is formulated as an anticancer agent for liver tumor treatment. We have designed a special NLC combined with an active ligand (GA) to achieve the desired targeting performance.

## 2. Results and Discussion

### 2.1. Synthesis and the Surface Characterization of GA-Phospholipid Derivative

**Scheme 1:**
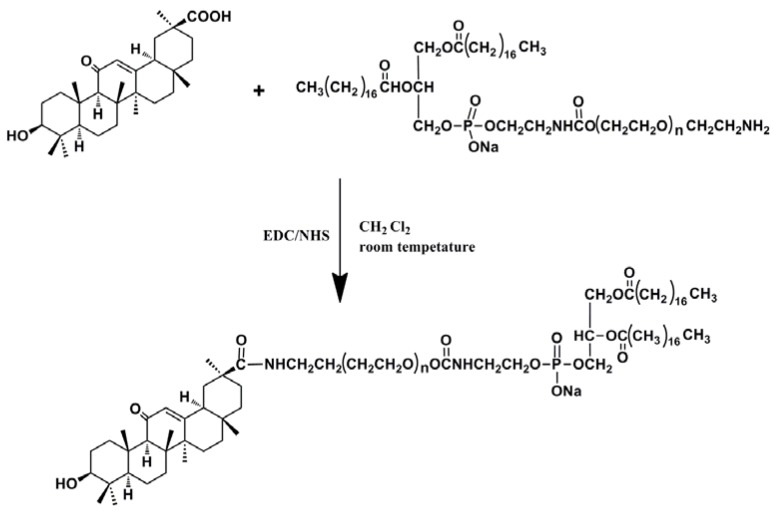
GA-PEG_2000_-DSPE reaction route.

The reaction steps are shown in Scheme 1.

#### 2.1.1. FT-IR

**Figure 3:**
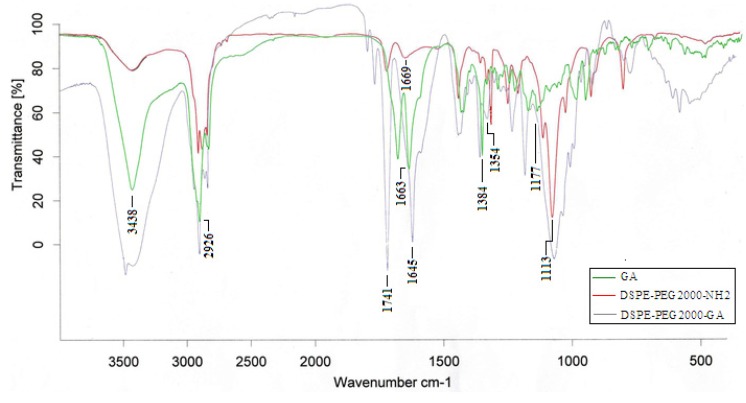
FT-IR spectra of GA, DSPE-PEG2000-NH2 and DSPE-PEG2000-GA.

Figure 3 shows the FT-IR spectra of GA, DSPE-PEG_2000_-NH_2_ and DSPE-PEG_2000_-GA polymer. The main resonance peaks of GA were -OH (3,438 cm^−1^), -CH_2_ (2,926 cm^−1^), -CH_3_ (1,384 cm^−1^), O-CH_2_ (1,177 cm^−1^) and C=C (1,663 cm^−1^), while the characteristic peaks of DSPE-PEG_2000_-NH_2_ were N-C=O (1,739 cm^−1^), -CH_2_ (2,917 cm^−1^), -CH_3_ (1,359 cm^−1^), O-CH_2_ (1,113 cm^−1^) and C=C (1,669 cm^−1^) respectively. The observed characteristic amide bond peak suggested that the targeted synthetic products were indeed formed. In the FT-IR spectrum of DSPE-PEG_2000_-GA, the appearance of characteristic peaks of 1,741 cm^−1^, 1,645 cm^−1^ and 1,354 cm^−1^ confirmed that the N-C=O bond was formed.

#### 2.1.2. 1H-NMR

**Figure 4:**
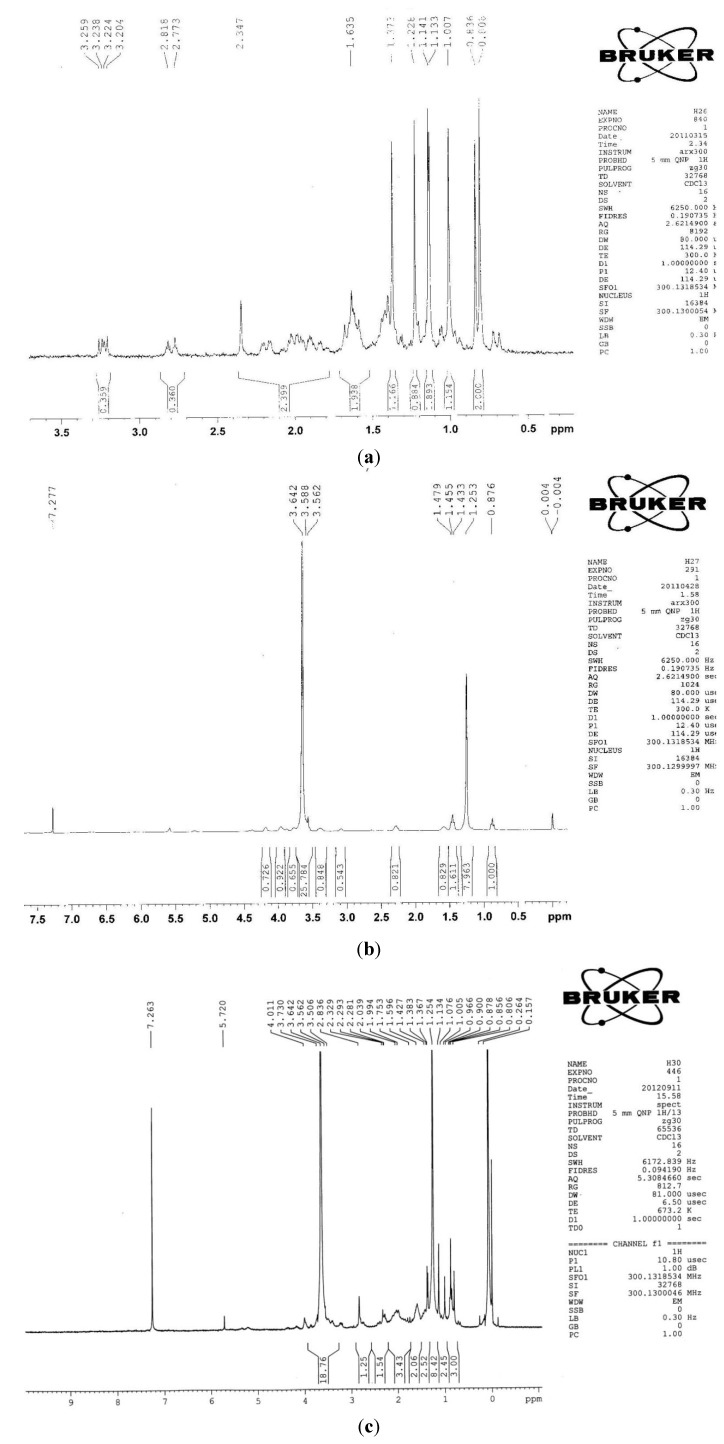
^1^H-NMR spectra of (**a**) GA; (**b**) DSPE-PEG2000-NH2; and (**c**) DSPE-PEG2000-GA.

Figure 4 shows the ^1^H-NMR spectra of GA, DSPE-PEG_2000_-NH_2_ and DSPE-PEG_2000_-GA polymer. The ^1^H-NMR spectrum of GA showed the methyl protons at δ 0.75–1.5 ppm. The ^1^H-NMR spectrum of DSPE-PEG_2000_-NH_2_ showed the characteristic -NH_2_ peak at δ 1.253 ppm, and the characteristic -CH_2_- peak at δ 3.64 ppm. The ^1^H-NMR spectrum of DSPE-PEG_2000_-GA contained characteristic peaks of GA (δ 0.75–1.5 ppm), -NH- (δ 1.254 ppm) and -CH_2_- (δ 3.64 ppm), indicating that the GA was successfully conjugated to DSPE-PEG_2000_.

### 2.2. Particle Characterization

**Figure 5:**
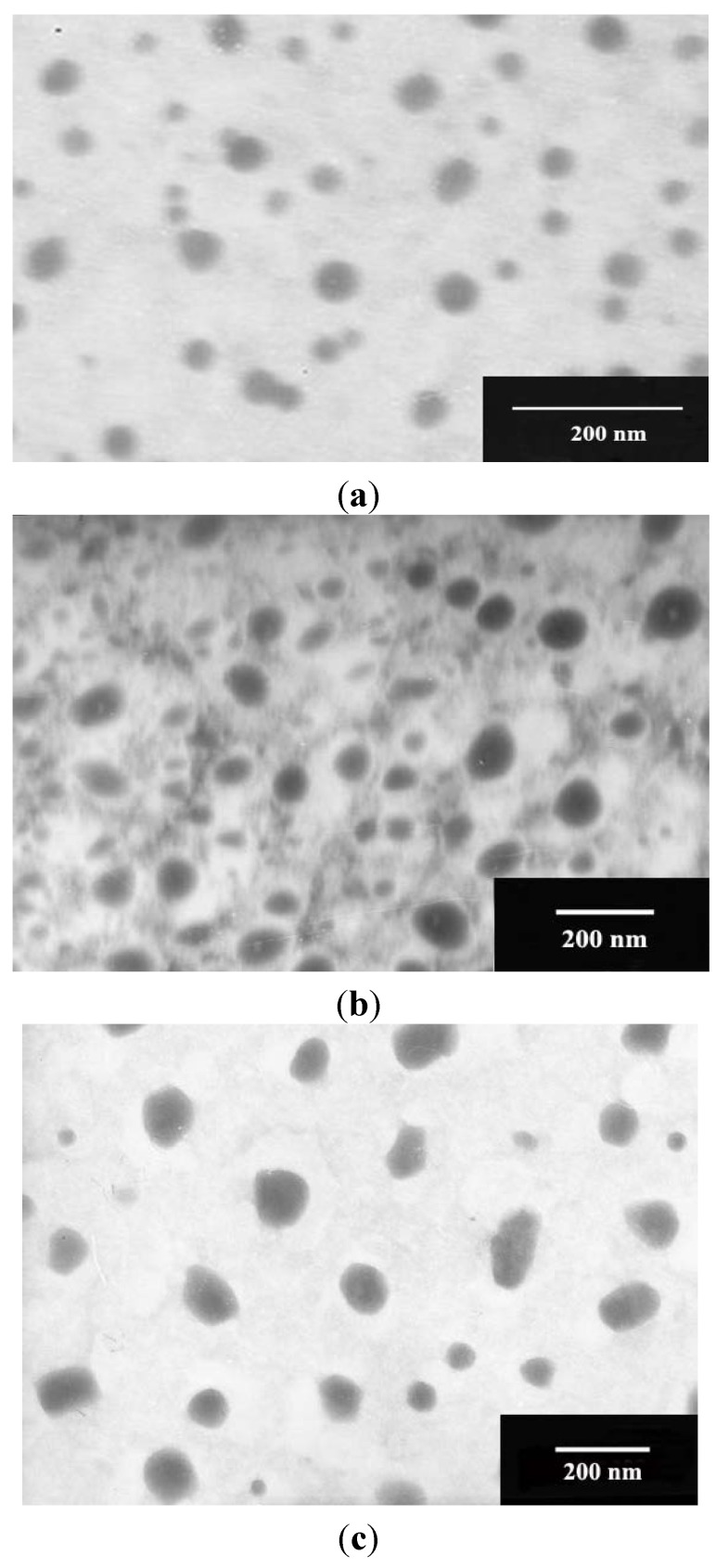
Transmission electron micrographs (TEM) of (**a**) Cur-NLC; (**b**) Cur-PEG-NLC; and (**c**) Cur-GA10%-PEG-NLC.

The Cur-NLC, Cur-PEG-NLC and Cur-GA-PEG-NLC formulation containing various concentrations of GA-modified PEG_2000_-DSPE (5%, 10% and 15%; w/w) were prepared by the film-ultrasonic technique. As shown in Figure 5, the appearance of Cur-NLC, Cur-PEG-NLC and Cur-GA10%-PEG-NLC particles was spherical or ellipsoidal with smooth surfaces. Compared with the Cur-NLC, Cur-PEG-NLC and Cur-GA10%-PEG-NLC have a larger particle size. The particle size, zeta potential, average encapsulation efficiency and the drug loading capacity results of Cur-NLC, Cur-PEG-NLC, Cur-GA5%-PEG-NLC, Cur-GA10%-PEG-NLC, Cur-GA15%-PEG-NLC are listed in Table 1.

**Table 1:** Particle characterization of Cur-NLC, Cur-PEG-NLC and Cur-GA5/10/15%-PEG-NLC (x ± SD, n = 5).

Type	Particle Characterization			
	Particle Sizes (nm)	Zeta Potentials (mV)	Encapsulation Efficiency (%)	Drug Loading Capacity (%)
Cur-NLC	58.3 ± 8.8	−22.6 ± 0.88	93.48 ± 0.86	2.25 ± 0.32
Cur-PEG-NLC	102.4 ± 13.5	−17.1 ± 0.53	97.12 ± 2.45	2.34 ± 0.28
Cur-GA5%-PEG-NLC	123.1 ± 15.6	−16.2 ± 0.48	95.31 ± 2.18	2.30 ± 0.30
Cur-GA10%-PEG-NLC	128.4 ± 15.9	−15.5 ± 0.37	93.11 ± 1.76	2.24 ± 0.22
Cur-GA15%-PEG-NLC	132.7 ± 16.7	−14.8 ± 0.32	90.06 ± 1.12	2.17 ± 0.24

### 2.3. Cellular Selective Uptake of NLCs

**Figure 6:**
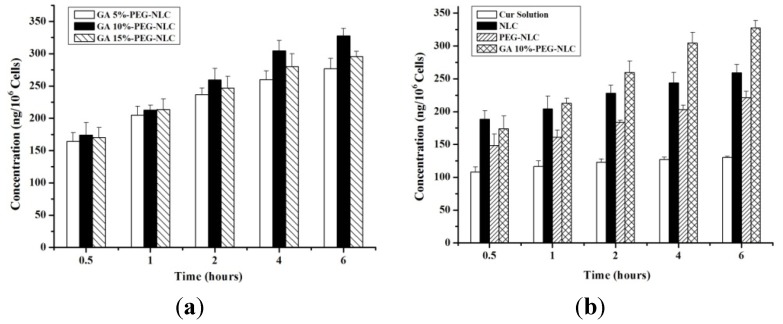
Cellular uptake content of (**a**) Cur-PEG-NLC with different GA ratio; and (**b**) Cur-Solution, NLC, PEG-NLC and GA10%-PEG-NLC.

Figure 6 showed the results of *in vitro* cellular uptake. Because Cur was trapped in the nanostructured lipid carrier and further internalized into tumor cells by endocytosis, cellular uptake of nanocarriers were higher than in the Cur-solution group. For the same concentration, the cellular uptake of free drug and the preparations were all increased over the incubation time. It was found that the cellular uptake reached a plateau after 6 h incubation, probably due to the saturation effect.

A series of the Cur-GA-PEG-NLCs with different GA ratio (5%, 10% and 15%; w/w) was prepared to investigate the influence of GA on the uptake of the preparations. The figure showed that the uptake was time dependent. At 0.5 h and 1 h, the three types of preparations had similar cell accumulation. However, after 2 h incubation, Cur-GA10%-PEG-NLC group had more cellular uptake than the other two groups. This result showed that the 10% of the GA on the surface of the nanocarrier was achieving the best cellular uptake because of the limited number of receptors on the cell surface. It was also reported that overabundance of ligands may block combination of receptor and ligand due to steric hindrance [27,28].

### 2.4. Cytotoxicity to HepG2

**Figure 7:**
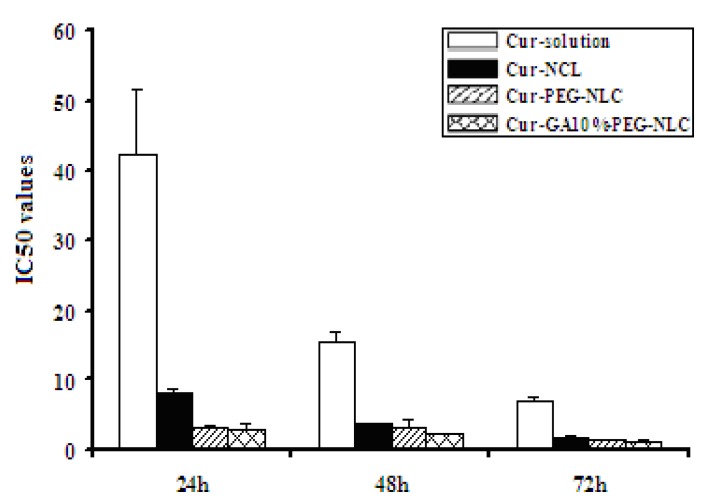
The IC_50_ values of of Cur-solution, Cur-NLC, Cur-PEG-NLC and Cur-GA10%-PEG-NLC (x ± SD, n = 4).

The IC_50_ values were listed in Table 2 and shown in Figure 7. Cytotoxicity test showed that the HepG_2_ cell proliferation inhibition rates of free drug and the formulations were concentration and time dependent. The cell growth inhibition rates were mostly increased with the increase in the time incubation and drug concentration. The group of Cur-GA10%-PEG-NLC had a much higher cytotoxicity than the group of Cur-solution at all the time points and higher than the group of Cur-NLC at 24, 48 h. 

**Table 2:** IC_50_ values of Cur-solution, Cur-NLC, Cur-PEG-NLC and Cur-GA10%-PEG-NLC (x ± SD, n = 4)

T (h)	IC_50_ (μg/mL)			
	Cur-Solution	Cur-NLC	Cur-PEG-NLC	Cur-GA10%-PEG-NLC
24	42.28 ± 9.40 **	8.00 ± 0.97 **	3.28 ± 0.16	2.91 ± 0.70
48	15.60 ± 1.28 **	3.87 ± 0.18 **	3.14 ± 1.27	2.22 ± 0.03
72	6.93 ± 0.61 **	1.70 ± 0.31	1.54 ± 0.04	1.34 ± 0.24

** *p* < 0.01 *vs.* the group of Cur-GA10%-PEG-NLC.

## 3. Experimental

### 3.1. General

Curcumin was purchased from J&K Scientific Co. (Beijing, China). A curcumin reference was supplied by the National Institutes for Food and Drug Control (Beijing, China). Glycerin monostearate was purchased from Bodi Chemical Co., Ltd. (Tianjin, China). Caprylic/capric acid glycerol ester (Miglyol 812N) was obtained from SASOL Co. (Hamburg, Germany). Polyoxyl (40) Stearate was supplied by Yi Weikang Biological Technology Co., Ltd. (Qingdao, China). Soybean lecithin for injection (PC > 95%) was obtained from Tywei Pharmaceutical Co., Ltd. (Shanghai, China). Dextrangel was purchased from Sino-American Biotechnology Co., Ltd. (Henan, China). DSPE-PEG_2000_-NH_2_ was obtained from Avanti Co. (Alabaster, MI, USA). Human liver hepatocellular carcinoma cell line (HepG_2_) was supplied by Queen & King Biological Chemical Co., Ltd. (Shanghai, China). All the other chemicals and reagents used were of analytical grade.

### 3.2. Preparation of Cur-NLC

Weighed amounts of lipid (290 mg of Glycerol monostearae/125 mg of Miglyol 812N/91 mg of lecithin) and curcumin (10 mg) were placed into a beaker, and heated up to 75 °C with a water bath. After dissolving the melt by adding anhydrous ethanol (5 mL), the organic solvent was removed using a rotary evaporator. A certain amount of surfactant (320 mg of Polyoxyethylene 40 Strearate) was dissolved in the sterile water with sonication and heating at 75 °C by water bath to produce a surfactant-containing aqueous solution. The aqueous phase was added into the oil phase at 75 °C with magnetic stirring for 5 min to prepare the primary emulsion. Redispersion while still hot by an ultrasonic cell crusher for 5 min and filtering through a 0.22 μm microporous membrane, the filtrate was replenished to 10 ml and solidified using an ice bath to obtain the curcumin-loaded nanostructured lipid carrier (Cur-NLC) dispersion.

### 3.3. Synthesis of GA-Phospholipid Derivative (GA-PEG_2000_-DSPE)

GA, DCC and NHS at a certain mole ratio (1.25:1:1) were dissolved in anhydrous methylene chloride (10 mL), and allowed to react for 3 h at room temperature. A DSPE-PEG_2000_-NH_2_ methylene chloride solution (5 mL, 0.2 mmol/mL) was added into the mixture. The whole synthesis step was performed under nitrogen protection and the mixture was allowed to react for 48 h at room temperature. After filtration of the final solution to remove the by-product, the target product was obtained by precipitating from the filtrate which was mixed with a suitable amount of ice-col ether aiming to isolate the residual GA. The precipitate was dissolved in DMF and dialyzed for 72 h. The solid product was obtained by freeze drying. 

### 3.4. Preparation of Cur-PEG-NLC and Cur-GA-PEG-NLC

According to the method of Section 3.2, Cur-PEG-NLC was prepared by adding 15% (w/w) of the total lipid amount of DSPE-PEG_2000_ to the oil phase. To prepare a series of Cur-GA-PEG-NLC formulations containing various concentration of GA-modified PEG_2000_-DSPE (5%, 10% and 15%; w/w), different amounts of GA-PEG_2000_-DSPE were used.

### 3.5. FT-IR and ^1^H-NMR Analysis

FT-IR spectra of GA, DSPE-PEG_2000-_NH_2_ and DSPE-PEG_2000_-GA were determined on a Bruker vector22 FTIR (Bruker, Zurich, Switzerland). These solid samples were mixed with KBr and pressed to a disk for measurement. A Bruker ARX-300 NMR was used to record the ^1^H-NMR spectra of the GA, DSPE-PEG_2000_-NH_2_ and DSPE-PEG_2000_-GA samples. CDCl_3_ was used as test solvent.

### 3.6. Measurement of Particles Size and Zeta Potential

Particle size and zeta potential of NLCs were assayed by a Zetasizer Nano 2S-90 (Malvern Instruments, Worcestershire, UK).

### 3.7. Transmission Electron Microscopy (TEM)

The morphology of NLCs were observed using TEM (JEM-1200EX, JEOL Co., Tokyo, Japan). An amount of NLCs was diluted to a suitable concentration with distilled water, placed on a copper grid and stained with 2% phosphotungstic acid solution. After drying at room temperature, the grid was examined by TEM.

### 3.8. Drug Encapsulation

The encapsulation efficiency of curcumin in the nanostructured lipid carriers (NLCs) was determined using the Sephadex G-50 mini-column centrifugation method [29]. The drug content was detected by a UV method. 

### 3.9. *In Vitro* Cellular Uptake and *In Vitro* Cytotoxicity

The HepG_2_ cell line was used to investigate cellular uptake of Cur-solution, Cur-NLC, Cur-PEG-NLC. Additionally, the effect of drug-loaded nanocarriers with different ligands on the cellular uptake was also evaluated. After routine culture of 0.5, 1, 2, 4 or 6 h, the cellular uptake was analyzed by fluorescence spectrophotometry (λ_ex_ = 471 nm, λ_em_ = 541 nm). All samples were analysed three times. 

The HepG_2_ cells were cultured in DMED medium supplemented with 10% FBS at 37 °C with 5% CO_2_ under fully humidified conditions. Cells were seeded onto a 96-well culture plate in 200 μL of culture medium. After incubation for 24 h, Cur-solution, Cur-NLC, Cur-PEG-NLC and Cur-GA10%-PEG-NLC at various concentrations were added into each well. The cells were cultured for additional 24, 48 or 72 h, followed by addition of 20 μL MTT solution (5 μg/mL) to each well for further incubation of 4 h. The supernatant was removed carefully. Then 150 μL of DMSO was added to each well and vibrated for 10 min until the formazan crystals were completely dissolved. The absorbance was measured by a TECAN SPECTRA instrument (TECAN Co., Männedorf, Switzerland).

IC_50_ software (RM6240) was used to calculate the IC_50_ values (concentration that inhibits cellular activity by 50% compared to that of the control group) of the free drug and the different curcumin- loaded nanocarrier formulations at 24, 48 or 72 h.

## 4. Conclusions

This research studied the preparation and the particle characterization of Cur-NLC, Cur-PEG-NLC and Cur-GA-PEG-NLC, and also studied their *in vitro* cellular uptake and *in vitro* cytotoxicity. Cur-GA-PEG-NLC has been successfully prepared by the film ultrasound method. Cur-GA-PEG-NLC displayed a spherical appearance, an optimal particle size and a high entrapment efficiency. The results of *in vitro* HepG_2_ cellular uptake showed that the GA-mediated nanostructured lipid carrier has the highest cellular uptake. Cellular uptake tendency at 6 h showed following order: Cur-GA10%-PEG-NLC > Cur-NLC > Cur-PEG-NLC > Cur-solution. Cur-GA-PEG-NLC also demonstrated an increased *in vitro* cytotoxicity compared to that of free drug solution against the HepG_2_ cell line. Pharmacokinetic and *in vivo* efficacy studies of Cur-GA-PEG-NLC will be conducted in future work. 
